# Translational autoregulation of *BZW1* and *BZW2* expression by modulating the stringency of start codon selection

**DOI:** 10.1371/journal.pone.0192648

**Published:** 2018-02-22

**Authors:** Gary Loughran, Andrew E. Firth, John F. Atkins, Ivaylo P. Ivanov

**Affiliations:** 1 School of Biochemistry and Cell Biology, University College Cork, Cork, Ireland; 2 Division of Virology, Department of Pathology, University of Cambridge, Cambridge, United Kingdom; 3 Department of Human Genetics, University of Utah, Salt Lake City, Utah, United States of America; 4 Laboratory of Gene Regulation and Development, Eunice Kennedy Shriver National Institute of Child Health and Human Development, National Institutes of Health, Bethesda, Maryland, United States of America; University of British Columbia, CANADA

## Abstract

The efficiency of start codon selection during ribosomal scanning in eukaryotic translation initiation is influenced by the context or flanking nucleotides surrounding the AUG codon. The levels of eukaryotic translation initiation factors 1 (eIF1) and 5 (eIF5) play critical roles in controlling the stringency of translation start site selection. The basic leucine zipper and W2 domain-containing proteins 1 and 2 (BZW1 and BZW2), also known as eIF5-mimic proteins, are paralogous human proteins containing C-terminal HEAT domains that resemble the HEAT domain of eIF5. We show that translation of mRNAs encoding BZW1 and BZW2 homologs in fungi, plants and metazoans is initiated by AUG codons in conserved unfavorable initiation contexts. This conservation is reminiscent of the conserved unfavorable initiation context that enables autoregulation of *EIF1*. We show that overexpression of BZW1 and BZW2 proteins enhances the stringency of start site selection, and that their poor initiation codons confer autoregulation on *BZW1* and *BZW2* mRNA translation. We also show that overexpression of these two proteins significantly diminishes the effect of overexpressing eIF5 on stringency of start codon selection, suggesting they antagonize this function of eIF5. These results reveal a surprising role for BZW1 and BZW2 in maintaining homeostatic stringency of start codon selection, and taking into account recent biochemical, genetic and structural insights into eukaryotic initiation, suggest a model for BZW1 and BZW2 function.

## Introduction

Eukaryotic translation initiation is complex and requires the activities of many factors [1, 2]. In eukaryotic translation initiation the small ribosome subunit, including the tRNA_i_^Met^ and assorted initiation factors constituting the 43S pre-initiation complex (PIC), binds to the mRNA 5’ cap and scans in the 3’ direction for an initiation codon. In most cases, initiation occurs at an AUG codon with favorable flanking nucleotides [3, 4]. The consensus initiation context in mammals is GCC(A/G)CC*AUG*G. The underlined nucleotides at positions −3 and +4 (relative to the +1 A of AUG, shown in italics) play the most important role [4] although the slight possibility that the +4 position effect could be an amino acid N-end rule effect [5] has yet to be definitively excluded. Although the consensus sequence varies to some extent from organism to organism and from one eukaryotic kingdom to another, the preference for purine at position -3 is nearly universal, as is selection against (and presumably unfavorability) of U at positions -6 to -1. Even though G at position +4 is preferred only in vertebrates and plants, selection against A at the same position is conserved in metazoa, fungi and plants [6, 7]

*In vitro* and *in vivo* work has shown that the PIC component, eIF1, is the critical player in discrimination between poor and optimal initiation contexts [8, 9]. eIF1 binds near the P-site on the 40S ribosomal subunit [10–12]. This results in the formation and maintenance of an ‘open’ conformation PIC favoring scanning [13]. Start codon recognition is followed by release of eIF1 from the PIC leading to a ‘closed’ conformation of the small subunit which favors initiation and prevents scanning [14, 15].

In *Saccharomyces cerevisiae*, mutations in *EIF1*, *EIF1A*, *EIF2*, *EIF3*, *EIF4G* and *EIF5*, which each encode factors associated with the PIC, affect start codon selection [14,15]. Until recently it was thought that the sole role of eIF5 is to promote hydrolysis of eIF2-bound GTP in response to start codon recognition [16,17]. It is now known that eIF5 also stabilizes the binding of GDP to eIF2 and acts to inhibit the GDP-GTP exchange function of eIF2β, i.e. it works as a guanine nucleotide dissociation inhibitor, or GDI [18]. There is additional evidence for a distinct role for eIF5 as a functional competitor to eIF1 for binding at a critical site in the small ribosomal subunit such that successful competition leads to ejection of eIF1 [14,19–21]. eIF1 dissociation would then stimulate the formation of the PIC in its closed conformation that favors initiation [22].

We previously showed that elevated levels of eIF1 in mammalian cells reduces utilization of poor context AUG codons and of non-AUG start codons [9]. Interestingly, the AUG initiation codon of eIF1 is flanked by poor context nucleotides in most eukaryotes for which sequence data is available [9,23]. In *S*. *cerevisiae*, overexpression of eIF1 led to reduced utilization of its own poor context AUG and also of non-AUG codons [24]. These and other experimental data are consistent with a model in which eIF1 levels are controlled by an autoregulatory mechanism where high eIF1 levels reduce translation initiation from its own mRNA’s start codon.

In another study we also showed that elevated eIF5 levels result in increased initiation at poor-context AUG codons and at non-AUG start codons [25]. Many eukaryotic mRNAs encoding eIF5 contain one or more inhibitory upstream open reading frames (uORFs) whose start codons are in conserved poor contexts. This suggested a model for autoregulation in which elevated eIF5 levels can increase initiation at these uORF start codons and consequently decrease translation from the *EIF5* main ORF start codon. Using a series of reporters initiated by either AUGs in different contexts or by non-AUG start codons, eIF5 and eIF1 overexpression were observed to have opposite and additive effects on the stringency of start codon selection. This suggests that eIF5 and eIF1 positively cross-regulate each other’s expression at the level of translation initiation, providing additional means for a homeostatic cellular control mechanism to maintain stringency in start codon selection [25].

Vertebrate BZW1 and BZW2 proteins (the latter is also known as 5MP1) contain C-terminal W2-type HEAT domains. Other proteins with this domain are the translation initiation factors eIF4GI (also known as EIF4G1), DAP5 (also known as EIF4G2), eIF4GII (also known as EIF4G3), eIF2Bε (EIF2B5) and eIF5. Of these, the similarity between the W2 HEAT domains of BZW and eIF5 is most notable [26], which has prompted some to refer to BZW2 as eIF5-mimic protein 1, 5MP1 [27]. It has been reported that BZW2 interacts with eIF2 and eIF3 and has weak GDI activity on eIF2 recycling [28]. Furthermore, strong interaction between BZW2 (5MP1) and eIF2 is responsible for the activation of ATF4 translation in response to BZW1 and BZW2 overexpression by sequestering the latter away from the ribosome [28]. Despite their homology to initiation factors and experiments implicating *BZW* orthologs in translation [27–31], the exact role of BZW1 and BZW2 in initiation is still under investigation.

Here we analyze sequences of *BZW* homologs throughout eukaryotes and demonstrate that, similar to *EIF1*, their start codons are in conserved poor context. We demonstrate that the poor initiation context, when present on luciferase reporters, directs lower initiation if BZW levels are high, implying autoregulation and implicating these proteins in the selection step of translation initiation.

## Materials and methods

### Sequence analysis

*BZW* homolog sequences were obtained from GenBank by tBLASTn with human BZW1 and BZW2 protein sequences as query. The sequences were derived from the Expressed Sequence Tag (EST), Transcriptome Shogun Assembly (TSA), Whole-Genome Shotgun contigs (WGS) or the RefSeq nucleotide databases. WGS sequences were processed manually to predict intron/exon junctions for the mRNA sequence. All alignments in this study were performed with the ClustalX algorithm [32]. Sequences used in this study are available upon request.

### Plasmids

Sense and antisense oligonucleotide pairs 1–2 and 3–4 (see S1 Table) were annealed and ligated into *Pst*I / *Bam*HI-digested dual luciferase vector p2-Luc [33] to make pSV40-firefly reporter constructs with the *BZW1* and *BZW2* initiation contexts respectively. The GUG initiated reporter was described previously [9]. BZW1-native and BZW1-optimal* were amplified by RT-PCR from RNA isolated from HEK-293T cells using sense primers 5 (native) and 6 (optimal*) and separately with antisense primer 7. BZW2-native and BZW2-optimal* were amplified by RT-PCR from RNA isolated from HEK-293T cells using sense primers 8 (native) and 9 (optimal*) and separately with antisense primer 10. Amplicons were cloned into *Nhe*I / *Xba*I-digested phRL-CMV (Promega). All constructs were verified by sequencing. The plasmid used to overexpress deregulated eIF1 (eIF1g*), and deregulated eIF5 (eIF5-AAA), have been described previously [9,25]. The plasmid used for control transfections was pcDNA3 (Invitrogen).

### Cell culture and transfections

HEK-293T cells (ATCC) were maintained in DMEM supplemented with 10% FBS, 1 mM L-glutamine and antibiotics. HEK-293T cells were transfected in quadruplicate with Lipofectamine 2000 reagent (Invitrogen), using the 1-day protocol in which suspended cells are added directly to the DNA complexes in half-area 96-well plates. For each transfection the following were added to each well: 50 ng of protein overexpressing vector (or 25 ng each for mixing experiments), 5 ng pSV40-firefly vector (with initiation contexts and/or codons as indicated in the figures), 0.2 ng pSV40-*Renilla* vector and 0.2 μl Lipofectamine 2000 in 25 μl Opti-Mem (Gibco). The transfecting DNA complexes in each well were incubated with 3 × 10^4^ cells suspended in 50 μl DMEM + 10% FBS. Transfected cells were incubated at 37°C in 5% CO_2_ for 16hr.

### Dual luciferase assay

Firefly and *Renilla* luciferase activities were determined using the Dual Luciferase Stop & Glo^®^ Reporter Assay System (Promega). Relative light units were measured on a Veritas Microplate Luminometer with two injectors (Turner Biosystems). Transfected cells were washed once with 1 × PBS and then lysed in 12.6 μl of 1 × passive lysis buffer (PLB) and light emission was measured following injection of 25 μl of either *Renilla* or firefly luciferase substrate. Firefly luciferase activity was calculated relative to the activity of the co-transfected control plasmid expressing *Renilla* luciferase (pSV40-*Renilla*).

### Western analysis

Cells were transfected in 6-well plates using Lipofectamine 2000 reagent, again using the 1-day protocol described above, with 1 μg of each indicated plasmid. The transfecting DNA complexes in each well were incubated with 8 × 10^5^ HEK-293T cells suspended in 3000 μl DMEM + 10% FBS and incubated overnight at 37°C in 5% CO_2_. Transfected cells were lysed in 100 μl 1 × PLB. Proteins were resolved by SDS-PAGE and transferred to nitrocellulose membranes (Protran), which were incubated at 4°C overnight with primary antibodies. Immunoreactive bands were detected on membranes after incubation with appropriate fluorescently labelled secondary antibody using a LI-COR Odyssey^®^ Infrared Imaging Scanner.

### Antibodies

The following commercially available antibodies were used: Rabbit anti-BZW1 (Abcam ab85090) diluted 1:500, rabbit anti-BZW2 (Abcam ab96682) diluted 1:1000, goat anti-eIF1 (Santa Cruz D-15) diluted 1:100, rabbit anti-eIF5 (Abcam ab85913) diluted 1:1000 and mouse anti-β-actin (Sigma A3853) diluted 1:5000.

## Results

### *BZW1* and *BZW2* display deeply conserved poor translation initiation context

**Fig 1:**
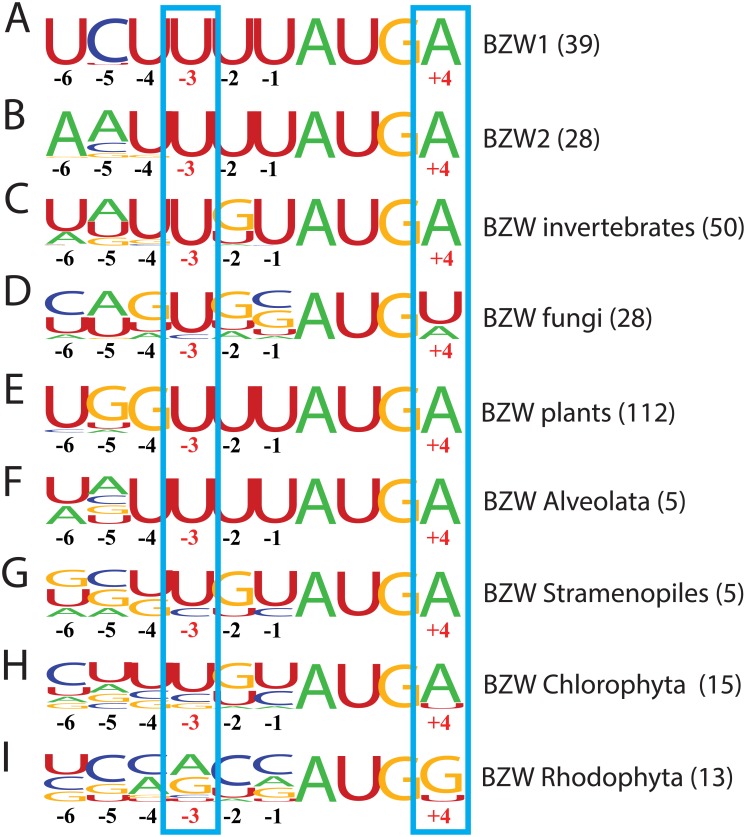
The AUG start codon of *BZW* homologs in eukaryotes is in conserved poor initiation context. The sequences surrounding the start codon of *BZW* homologs, from position −6 to +4, were aligned for the evolutionary branch indicated and the alignment represented as a frequency logo [34], with the height of each letter proportional to the frequency with which the corresponding nucleotide occurs at a given position. Alignment of: (A) *BZW1* orthologs in vertebrates; (B) *BZW2* orthologs in vertebrates; (C) *BZW* homologs in invertebrates; (D) *BZW* homologs in fungi; (E) *BZW* homologs in land plants; (F) *BZW* homologs in Alveolata; (G) *BZW* homologs in brown algae; (H) *BZW* homologs in green algae; and (I) *BZW* homologs in red algae. The critical positions −3 and +4 are indicated with red numbers and also boxed. Numbers in parentheses indicate the number of sequences in each alignment.

Following up on the translational autoregulation observed with *EIF1* [9] and *EIF5* [25], which both exploit conserved poor translation initiation context, we decided to perform a search for other human genes with clear patterns of conserved poor context surrounding their initiation codons. This search identified a pair of human paralogs called *BZW1* and *BZW2*, with poor initiation context displaying deep evolutionary conservation (Fig 1, S1 Fig). Previously, the amino acid sequences of 97 eukaryotic *BZW* homologs were examined [30] and here we extend this analysis by comparing partial or complete nucleotide sequences of 250 eukaryotic *BZW* homologs. *BZW* homologs are present in metazoa, some fungi (though *BZW* orthologs are not apparent in Ascomycota, including *S*. *cerevisiae*), plants, green algae (Chlorophyta), red algae (Rhodophyta), brown algae (Stramenopiles), and also in some protists (e.g. Alveolata). The initiation context of the first in-frame AUG of *BZW* homologs from animals, plants, fungi, Alveolata, and brown algae is present in conserved poor context (Fig 1). A purine is never observed at position −3, but rather the least favorable nucleotide, U, and on rare occasions the almost equally unfavorable C are found at that position (Fig 1A–1G). Furthermore, there is never a G at position +4 and there is a preponderance of unfavorable U-s at positions −6, −4, −2 and −1 in many evolutionary branches. In green algae the −3 position is predominately unfavorable U or C. Although there is no apparent selection for poor initiation context in red algae, overall the selection for poor context at positions −3 and +4 is comparable to that observed in *EIF1*, where the poor context has been shown to be essential for its autoregulation [9].

In humans, *BZW1* and *BZW2* contain 3 and 4 out-of-frame AUG codons, respectively, between the first and second in-frame AUG codons of the main ORF. In each case at least two of these out-of-frame AUG codons are present in intermediate or better initiation context (S2 Fig). Following the principles of leaky scanning it would be expected that any 43S subunits which scan past the first in-frame AUG codon would likely be “captured” by one of the out-of-frame AUG codons, so that only an insignificant fraction of 43S subunits would ever reach the second in-frame AUG. This ORF architecture, which is also common in BZW homologs in other eukaryotes, suggests that the first in-frame AUG is the sole initiation codon in both *BZW1* and *BZW2* mRNAs, and that the purpose of the conserved weak initiator codon in *BZW1* and *BZW2* is not to generate one of several isoforms by leaky scanning, as is observed in some other mRNAs in which the first in-frame AUG is in poor context [35,36].

### Overexpression of BZW1 leads to higher initiation stringency, which is autoregulatory

If the conserved *BZW1* and *BZW2* initiation sites are sensors for negative feedback regulation, then initiation from these sites should be inefficient under control conditions and should become increasingly so as the intracellular concentration of BZW1 and BZW2 increase.

**Fig 2:**
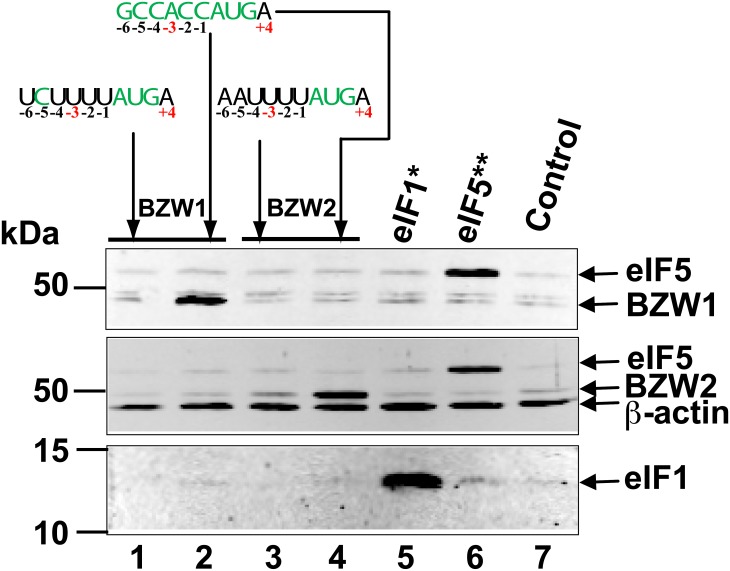
*BZW1* and *BZW2* are expressed poorly from their native initiation context. Anti-BZW immunoblots of lysates prepared from HEK-293T cells transfected with BZW-expressing plasmids with start codons in different contexts as indicated. *BZW1* in native context (lane 1), *BZW1* in optimal context (lane 2), *BZW2* in native context (lane 3), *BZW2* in optimal context (lane 4). Overexpression of deregulated stringency factors eIF1 and eIF5 are shown in lanes 5 and 6 respectively. The control lane shows lysates from cells transfected with pcDNA3.

The prediction that *BZW1* and *BZW2* contexts are poor were tested by cotransfecting HEK-293T cells with plasmids expressing human *BZW1* or *BZW2* initiated by AUG in either native (predicted to be poor) or optimal initiation context (as defined by Kozak) (Fig 2). Western blots from lysates of these transfected cells confirm that BZW1 and BZW2 proteins overexpressed from plasmids with *BZW1* and *BZW2* native initiation contexts are inefficiently expressed compared to the same proteins initiated by AUG in context optimized at positions −6 to −1 (compare lane 1 to lane 2 for BZW1 and lane 3 to lane 4 for BZW2 in Fig 2).

**Fig 3:**
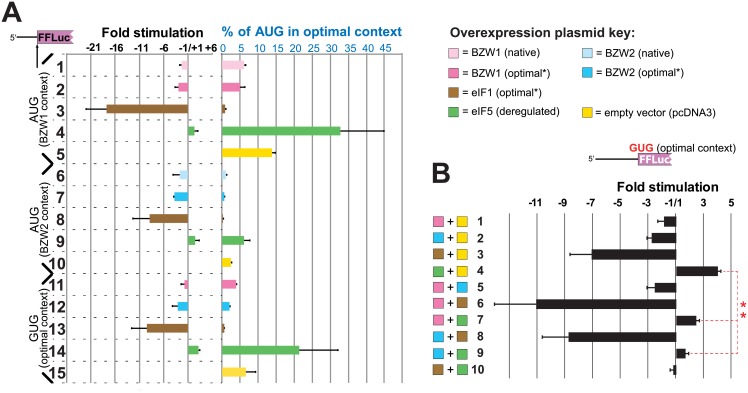
Overexpression of BZW1 and BZW2 leads to higher stringency of start codon selection and cancels the effect of eIF5 overexpression. (A) Dual luciferase assays showing fold stimulation (left-hand side) or percentage initiation relative to AUG in optimal context (right-hand side) of firefly luciferase reporters initiated with start codons in native BZW contexts or with GUG as indicated (left-hand margin) in response to overexpression of the indicated initiation factors. (B) Dual luciferase assays showing fold stimulation of GUG-initiated firefly luciferase reporters in response to overexpression of combinations of the indicated initiation factors. For both (A) and (B), firefly luciferase measurements were normalized to those from a co-transfected *Renilla* luciferase expressing construct. For the fold stimulation calculations, the ratios in test cells were compared to the luciferase ratio in control cells transfected with pcDNA3. Negative "stimulation" values indicate repression. “Optimal*” indicates initiation context which is optimized at nucleotide positions −6 to −1 relative to the start codon, but not at position +4 which is kept the same as the native sequence to prevent changes in the encoded amino acid. **p<0.001 (Student’s two-tailed *t*-test, n = 6).

Initiation of reporters starting with the native *BZW1* and *BZW2* AUG contexts was measured using firefly luciferase reporters relative to reporters starting with AUG in optimal context. The level of initiation at the *BZW1* and *BZW2* start codons when fused to firefly luciferase is ~13% and ~2.5% of an optimal context AUG, respectively (see 5 and 10 in Fig 3A). Although both AUGs are in poor context, the much lower level of expression from the *BZW2* AUG likely underlies the importance of minor context determinants when there is neither a purine at −3 nor a G at +4 (see Fig 1).

Overexpression of *BZW1* (with native AUG context) results in a 2.3 fold decrease in BZW1-reporter expression compared to cells transfected with empty vector (compare 1 and 5 Fig 3A). A further slight, but not statistically significant, decrease in firefly luciferase activity is observed when cells overexpress *BZW1* that has been deregulated by optimizing its AUG context at positions −6 to −1 (compare 1, 2 and 5 in Fig 3A). Overexpression of *BZW2* has a similar effect on reporters starting with AUG in the native *BZW2* context (compare lanes 6, 7 and 10 in Fig 3A). Together, these data indicate that BZW1 and BZW2 can suppress initiation of their own start codons, and by implication they can autoregulate their own expression.

To assess the effect of BZW1 and BZW2 levels on overall stringency we also tested reporter constructs initiated by GUG in the presence of overexpressed *BZW1* or *BZW2* (with optimized context at positions −6 to −1) (compare lanes 11, 12 and 15 in Fig 3A). Since we know that both eIF1 and eIF5 can regulate initiation at suboptimal translation start codons we also separately overexpressed each of these initiation factors (see 3, 4, 8, 9, 13 and 14 in Fig 3A). These results indicate that the effect of *BZW1* or *BZW2* overexpression is not restricted to their native initiation context but leads to an overall increase in stringency of start codon selection on both AUGs in poor context and on near-cognate start codons.

### Overexpression of BZW inhibits the effect of overexpressing eIF5, suggesting the two have an antagonistic relationship in setting the stringency of start codon selection

**Fig 4:**
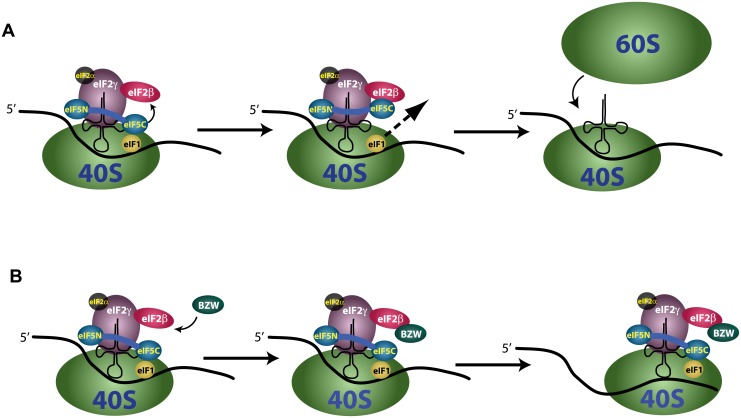
A model for the molecular interactions likely to account for the effect of BZW1 and BZW2 on stringency of start codon selection. During scanning, when the 40S subunit is in the open conformation, eIF1 is bound near the P-site and monitors the interaction between tRNA_i_^Met^ and nucleotide triplets in the mRNA. The eIF5 C-terminal domain is loosely bound to eIF1 working as a lid to prevent the premature release of eIF1 from the PIC. (A) Under low BZW levels, the eIF2β N-terminal domain and eIF5 C-terminal domain occasionally interact pulling eIF5 away from eIF1, allowing its looser association with the PIC. The lower affinity of eIF1 for the PIC facilitates more frequent initiation at near-cognate or suboptimal AUG codons. (B) BZW, perhaps through its C-terminal HEAT domain, binds to the eIF2β N-terminal domain. When levels of BZW protein are high this interaction is favored, and this prevents interaction between eIF2β and eIF5. This leaves the “lid” on eIF1 closed, essentially increasing its effective concentration on the PIC and this enhances stringency of start codon selection, resulting in lower frequency of initiation at near-cognate start codons or poor context AUG triplets, including the start codons of BZW genes, with ribosome continuing to scan in the 3’ direction for a better start codon.

As discussed above, BZW2 and also BZW1 are thought to act as eIF5-mimic proteins by interfering with ternary complex (TC) recruitment to the 40S subunit. At the same time, high concentrations of eIF5 lead to ejection of eIF1 from the PIC and induce a closed conformation of the 40S subunit. If BZW1 or BZW2 can block this latter activity of eIF5, their overexpression would be expected to increase stringency by preventing ejection of eIF1 from the PIC even in the presence of high levels of eIF5, irrespective of their earlier role in PIC recruitment. To test this hypothesis we transfected constructs expressing BZW1 or BZW2 together with either eIF1 or eIF5 along with firefly luciferase reporters initiated with GUG (Fig 3B). Overexpression of BZW1, BZW2 or eIF1 alone (plus equivalent amounts of empty vector) causes ~1.5, ~3 and ~7 fold decreases in initiation of reporters starting with GUG, respectively (1–3 in Fig 3B). Elevated levels of eIF5, by contrast, leads to ~4 fold increase in expression of the same reporter (4 in Fig 3B). When BZW1 or BZW2 are co-transfected with eIF5, the reduction of stringency seen with overexpression of eIF5 alone is significantly diminished, from ~4 fold with eIF5 alone to either ~2.5 fold (with co-overexpression of BZW1) or ~0.75 fold (with co-overexpression of BZW2) (7 and 9 in Fig 3B). An additive effect for co-transfection of BZW1 or BZW2 along with eIF1 is less clear (6 and 8 in Fig 3B). These results indicate that BZW1 and BZW2 can affect the stringency of start codon selection by reducing the actions of eIF5. Given the homology of BZW1 and BZW2 with eIF5 and the findings of Nanda and colleagues [14,19], we suggest that BZW1 and BZW2 act as dominant negative inhibitors of eIF5 as shown in the model (Fig 4).

## Discussion

The results presented above provide strong evidence that elevated levels of BZW1 and BZW2 increase the stringency of start codon selection in mammalian cells. They also suggest that this is used in autoregulation of BZW1 and BZW2 expression in most if not all eukaryotes that possess orthologs of these genes. A central feature of this autoregulation is the presence of an obligatory AUG start codon in unfavorable initiation context. The suboptimal initiation contexts of human *BZW1* and *BZW2* was established by western analysis of BZW1 and BZW2 levels synthesised from plasmids where they are initiated by their native or by optimal context (Fig 2). High levels of BZW1 and BZW2 were shown to result in greater discrimination against these start codons lowering the synthesis of firefly luciferase reporters starting with *BZW1* and *BZW2* initiation contexts (Fig 3A). The results also show that high levels of BZW1 and BZW2 by themselves increase the stringency of start codon selection and significantly neutralize the effect of overexpressing eIF5, which by itself lowers the stringency of start codon selection (Fig 3).

The precise molecular mechanism of start codon selection is gradually coming into focus. Several initiation factors have been identified genetically and biochemically that provide interactions implicated in this process. These include eIF1, eIF1A, eIF5, eIF2, eIF3 and eIF4G [2,37]. With two of them, eIF1 and eIF5, it has been shown that their protein levels in the cell directly affect stringency of start codon selection, with high levels of eIF1 increasing stringency and high levels of eIF5 relaxing it [9,24,25,38,39]. In both of these cases, as is shown here for BZW1 and BZW2, this is used for autoregulation [9,24,25]. eIF1 promotes high stringency by binding 40S ribosomes to facilitate the open scanning complex [12, 13]. Its release, upon tRNA_i_^Met^ encountering a favorable start codon, leads to a closed PIC, which precludes further scanning [14,40]. The exact molecular mechanism by which high levels of eIF5 reduce stringency are not known, however, high concentrations of eIF5 help eject eIF1 from a PIC which has encountered a suboptimal start codon *in vitro* [14,19]. It has been proposed that eIF5 has a lower affinity binding site on the 40S subunit and that this site overlaps the binding site of eIF1 [14]. In addition, the eIF5 C-terminal domain (CTD], encoding its HEAT domain, interacts with eIF1 and this interaction is involved in start codon recognition [20], and it is the CTD domain of eIF5 that antagonizes the binding of eIF1 to the PIC [19]. Separately, the eIF5 CTD interacts with the N-terminal domain (NTD) of eIF2β [20], and also with eIF3c and eIF4G [41]. The surfaces on the eIF5 CTD that bind to eIF1 and eIF2β partially overlap, implying mutually exclusive interactions. It is believed that the interaction of the eIF5 CTD with eIF1 impedes the premature release of the latter during scanning. Conversly, the interaction between the NTD of eIF2β and the CTD of eIF5 is believed to separate eIF5 from eIF1, thus facilitating the release of eIF1 [20]. Immunoprecipitation experiments suggest interaction between BZW and eIF2β [27]. The precise locations of the eIF5 CTD and the eIF2β NTD are not defined in any of the published structures for the PIC, implying that they are afforded considerable flexibility. Here we show that overexpression of either BZW1 or BZW2 increases stringency of start codon selection and this might be synergistic with the activity of eIF1 and is antagonistic with the activity of eIF5. Based on these data, and the published results outlined above, we propose the following model for the role of BZW proteins in start codon selection (Fig 4): During scanning, when the 40S subunit is in the open conformation, eIF1 is bound near the P-site and monitors the interaction between tRNA_i_^Met^ and nucleotide triplets in the mRNA. The eIF5 CTD is loosely bound to eIF1 working as a lid to prevent the premature release of eIF1 from the PIC. Under low BZW levels, the eIF2β NTD and eIF5 CTD occasionally interact pulling eIF5 away from eIF1, allowing its looser association with the PIC and facilitating more frequent initiation at near-cognate or suboptimal AUG codons. When BZW levels are high the protein, perhaps through its CTD HEAT domain, binds to the eIF2β NTD and prevents its interaction with eIF5. This leaves the “lid” on eIF1 closed and enhances stringency of start codon selection.

Given the known interactions of eIF2β, eIF1, eIF5, BZW1 and BZW2 with the 40S subunit we prefer a model where these competitive interactions involve the PIC but competition in solution cannot be ruled out.

## Supporting information

S1 Fig Conservation of the initiation context of *BZW* homologs. Sequences used for generating the logograms in Fig 1. Species identifiers of the sequences used are indicated on the left. Start codons are highlighted in green. Numbers in parentheses indicate the number of sequences in each phylogenetic branch.
(PDF)

S2 Fig The nucleotide sequences of human *BZW1* and *BZW2* precludes the likelihood of leaky scanning producing N-terminally truncated proteins. (A) The sequence of human *BZW1* mRNA. (B) The sequence of human *BZW2* mRNA. The main open reading frame is highlighted in yellow. The stop codon is highlighted in red. The first and second in-frame AUG codons are highlighted in light green. The out-of-frame AUG codons between the first and second in-frame AUG codons are highlighted in dark green. −3 and +4 nucleotides matching favorable initiation context are highlighted in gray, while those that are unfavorable are highlighted in magenta.
(PDF)

S1 Table DNA oligonucleotides used in this study. (PDF)

## References

[1] Hinnebusch AG, Dever TE, Asano K. Mechanism of translation initiation in the yeast Saccharomyces cerevisiae In Translational Control in Biology and Medicine. Mathews M, Sonenberg N, Hershey JWB, editors. Cold Spring Harbor; 2007.

[2] Jackson RJ, Hellen CUT, Pestova T V. The mechanism of eukaryotic translation initiation and principles of its regulation. Nat Rev Mol Cell Biol. 2010;11: 113–127. doi: 10.1038/nrm2838 20094052 10.1038/nrm2838 PMC4461372

[3] Kozak M. Compilation and analysis of sequences upstream from the translational start site in eukaryotic mRNAs. Nucleic Acids Res. 1984;12: 857–72. Available: http://www.ncbi.nlm.nih.gov/pubmed/6694911 6694911 10.1093/nar/12.2.857 PMC318541

[4] Kozak M. Point mutations define a sequence flanking the AUG initiator codon that modulates translation by eukaryotic ribosomes. Cell. 1986;44: 283–292. doi: 10.1016/0092-8674(86)90762-2 3943125 10.1016/0092-8674(86)90762-2

[5] Varshavsky A. The N-end rule pathway of protein degradation. Genes Cells. 1997;2: 13–28. Available: http://www.ncbi.nlm.nih.gov/pubmed/9112437 9112437 10.1046/j.1365-2443.1997.1020301.x

[6] Nakagawa S, Niimura Y, Gojobori T, Tanaka H, Miura K. Diversity of preferred nucleotide sequences around the translation initiation codon in eukaryote genomes. Nucleic Acids Res. Oxford University Press; 2008;36: 861–71. doi: 10.1093/nar/gkm1102 18086709 10.1093/nar/gkm1102 PMC2241899

[7] Jacobs GH, Chen A, Stevens SG, Stockwell PA, Black MA, Tate WP, et al Transterm: a database to aid the analysis of regulatory sequences in mRNAs. Nucleic Acids Res. Oxford University Press; 2009;37: D72–6. doi: 10.1093/nar/gkn763 18984623 10.1093/nar/gkn763 PMC2686486

[8] Pestova T V., Kolupaeva VG. The roles of individual eukaryotic translation initiation factors in ribosomal scanning and initiation codon selection. Genes Dev. 2002;16: 2906–2922. doi: 10.1101/gad.1020902 12435632 10.1101/gad.1020902 PMC187480

[9] Ivanov IP, Loughran G, Sachs MS, Atkins JF. Initiation context modulates autoregulation of eukaryotic translation initiation factor 1 (eIF1). Proc Natl Acad Sci U S A. 2010;107: 18056–18060. doi: 10.1073/pnas.1009269107 20921384 10.1073/pnas.1009269107 PMC2964218

[10] Lomakin IB, Kolupaeva VG, Marintchev A, Wagner G, Pestova T V. Position of eukaryotic initiation factor eIF1 on the 40S ribosomal subunit determined by directed hydroxyl radical probing. Genes Dev. 2003;17: 2786–2797. doi: 10.1101/gad.1141803 14600024 10.1101/gad.1141803 PMC280627

[11] Rabl J, Leibundgut M, Ataide SF, Haag A, Ban N. Crystal structure of the eukaryotic 40S ribosomal subunit in complex with initiation factor 1. Science. 2011;331: 730–6. doi: 10.1126/science.1198308 21205638 10.1126/science.1198308

[12] Hinnebusch AG. Structural Insights into the Mechanism of Scanning and Start Codon Recognition in Eukaryotic Translation Initiation. Trends Biochem Sci. 2017; doi: 10.1016/j.tibs.2017.03.004 28442192 10.1016/j.tibs.2017.03.004

[13] Passmore LA, Schmeing TM, Maag D, Applefield DJ, Acker MG, Algire MA, et al The eukaryotic translation initiation factors eIF1 and eIF1A induce an open conformation of the 40S ribosome. Mol Cell. 2007;26: 41–50. doi: 10.1016/j.molcel.2007.03.018 17434125 10.1016/j.molcel.2007.03.018

[14] Nanda JS, Cheung YN, Takacs JE, Martin-Marcos P, Saini AK, Hinnebusch AG, et al eIF1 Controls Multiple Steps in Start Codon Recognition during Eukaryotic Translation Initiation. J Mol Biol. 2009;394: 268–285. doi: 10.1016/j.jmb.2009.09.017 19751744 10.1016/j.jmb.2009.09.017 PMC2783965

[15] Hinnebusch AG. The scanning mechanism of eukaryotic translation initiation. Annu Rev Biochem. 2014;83: 779–812. doi: 10.1146/annurev-biochem-060713-035802 24499181 10.1146/annurev-biochem-060713-035802

[16] Das S, Ghosh R, Maitra U. Eukaryotic Translation Initiation Factor 5 Functions as a GTPase-activating Protein. J Biol Chem. 2001;276: 6720–6726. doi: 10.1074/jbc.M008863200 11092890 10.1074/jbc.M008863200

[17] Paulin FE, Campbell LE, O’Brien K, Loughlin J, Proud CG. Eukaryotic translation initiation factor 5 (eIF5) acts as a classical GTPase-activator protein. Curr Biol. 2001;11: 55–9. Available: http://www.ncbi.nlm.nih.gov/pubmed/11166181 11166181 10.1016/s0960-9822(00)00025-7

[18] Jennings MD, Pavitt GD. eIF5 has GDI activity necessary for translational control by eIF2 phosphorylation. Nature. 2010;465: 378–381. doi: 10.1038/nature09003 20485439 10.1038/nature09003 PMC2875157

[19] Nanda JS, Saini AK, Munoz AM, Hinnebusch AG, Lorsch JR. Coordinated Movements of Eukaryotic Translation Initiation Factors eIF1, eIF1A, and eIF5 Trigger Phosphate Release from eIF2 in Response to Start Codon Recognition by the Ribosomal Preinitiation Complex. J Biol Chem. 2013;288: 5316–5329. doi: 10.1074/jbc.M112.440693 23293029 10.1074/jbc.M112.440693 PMC3581429

[20] Luna RE, Arthanari H, Hiraishi H, Nanda J, Martin-Marcos P, Markus MA, et al The C-terminal domain of eukaryotic initiation factor 5 promotes start codon recognition by its dynamic interplay with eIF1 and eIF2β. Cell Rep. 2012;1: 689–702. doi: 10.1016/j.celrep.2012.04.007 22813744 10.1016/j.celrep.2012.04.007 PMC3401385

[21] Obayashi E, Luna RE, Nagata T, Martin-Marcos P, Hiraishi H, Singh CR, et al Molecular Landscape of the Ribosome Pre-initiation Complex during mRNA Scanning: Structural Role for eIF3c and Its Control by eIF5. Cell Rep. NIH Public Access; 2017;18: 2651–2663. doi: 10.1016/j.celrep.2017.02.052 28297669 10.1016/j.celrep.2017.02.052 PMC5382721

[22] Asano K, Sachs MS. Translation factor control of ribosome conformation during start codon selection. Genes Dev. Cold Spring Harbor Laboratory Press; 2007;21: 1280–7. doi: 10.1101/gad.1562707 17545463 10.1101/gad.1562707

[23] Miyasaka H, Endo S, Shimizu H. Eukaryotic translation initiation factor 1 (eIF1), the inspector of good AUG context for translation initiation, has an extremely bad AUG context. J Biosci Bioeng. 2010;109: 635–7. doi: 10.1016/j.jbiosc.2009.11.022 20471606 10.1016/j.jbiosc.2009.11.022

[24] Martin-Marcos P, Cheung Y-N, Hinnebusch AG. Functional Elements in Initiation Factors 1, 1A, and 2 Discriminate against Poor AUG Context and Non-AUG Start Codons. Mol Cell Biol. 2011;31: 4814–4831. doi: 10.1128/MCB.05819-11 21930786 10.1128/MCB.05819-11 PMC3232919

[25] Loughran G, Sachs MS, Atkins JF, Ivanov IP. Stringency of start codon selection modulates autoregulation of translation initiation factor eIF5. Nucleic Acids Res. 2012;40: 2898–2906. doi: 10.1093/nar/gkr1192 22156057 10.1093/nar/gkr1192 PMC3326321

[26] Aravind L, Koonin E V. Eukaryote-specific domains in translation initiation factors: implications for translation regulation and evolution of the translation system. Genome Res. Cold Spring Harbor Laboratory Press; 2000;10: 1172–84. Available: http://www.ncbi.nlm.nih.gov/pubmed/10958635 10.1101/gr.10.8.1172 PMC310937 10958635

[27] Singh CR, Watanabe R, Zhou D, Jennings MD, Fukao A, Lee B, et al Mechanisms of translational regulation by a human eIF5-mimic protein. Nucleic Acids Res. 2011;39: 8314–8328. doi: 10.1093/nar/gkr339 21745818 10.1093/nar/gkr339 PMC3201852

[28] Kozel C, Thompson B, Hustak S, Moore C, Nakashima A, Singh CR, et al Overexpression of eIF5 or its protein mimic 5MP perturbs eIF2 function and induces *ATF4* translation through delayed re-initiation. Nucleic Acids Res. 2016;44: 8704–8713. doi: 10.1093/nar/gkw559 27325740 10.1093/nar/gkw559 PMC5062967

[29] Lee S, Nahm M, Lee M, Kwon M, Kim E, Zadeh AD, et al The F-actin-microtubule crosslinker Shot is a platform for Krasavietz-mediated translational regulation of midline axon repulsion. Development. 2007;134: 1767–1777. doi: 10.1242/dev.02842 17409115 10.1242/dev.02842

[30] Hiraishi H, Oatman J, Haller SL, Blunk L, McGivern B, Morris J, et al Essential role of eIF5-mimic protein in animal development is linked to control of ATF4 expression. Nucleic Acids Res. Oxford University Press; 2014;42: 10321–10330. doi: 10.1093/nar/gku670 25147208 10.1093/nar/gku670 PMC4176352

[31] Tang L, Morris J, Wan J, Moore C, Fujita Y, Gillaspie S, et al Competition between translation initiation factor eIF5 and its mimic protein 5MP determines non-AUG initiation rate genome-wide. Nucleic Acids Res. 2017;45: 11941–11953. doi: 10.1093/nar/gkx808 28981728 10.1093/nar/gkx808 PMC5714202

[32] Larkin MA, Blackshields G, Brown NP, Chenna R, McGettigan PA, McWilliam H, et al Clustal W and Clustal X version 2.0. Bioinformatics. 2007;23: 2947–2948. doi: 10.1093/bioinformatics/btm404 17846036 10.1093/bioinformatics/btm404

[33] Grentzmann G, Ingram JA, Kelly PJ, Gesteland RF, Atkins JF. A dual-luciferase reporter system for studying recoding signals. RNA. 1998;4: 479–486. 9630253 PMC1369633

[34] Crooks GE, Hon G, Chandonia JM, Brenner SE. WebLogo: A sequence logo generator. Genome Res. 2004;14: 1188–1190. doi: 10.1101/gr.849004 15173120 10.1101/gr.849004 PMC419797

[35] Kozak M. Pushing the limits of the scanning mechanism for initiation of translation. Gene. 2002 pp. 1–34. doi: 10.1016/S0378-1119(02)01056-9 10.1016/S0378-1119(02)01056-9 PMC7126118 12459250

[36] Bazykin GA, Kochetov A V. Alternative translation start sites are conserved in eukaryotic genomes. Nucleic Acids Res. 2011;39: 567–577. doi: 10.1093/nar/gkq806 20864444 10.1093/nar/gkq806 PMC3025576

[37] Hinnebusch AG. Molecular Mechanism of Scanning and Start Codon Selection in Eukaryotes. Microbiol Mol Biol Rev. 2011;75: 434–467. doi: 10.1128/MMBR.00008-11 21885680 10.1128/MMBR.00008-11 PMC3165540

[38] Barth-Baus D, Bhasker CR, Zoll W, Merrick WC. Influence of translation factor activities on start site selection in six different mRNAs. Translation. 2013;1: e24419 doi: 10.4161/trla.24419 26824019 10.4161/trla.24419 PMC4718060

[39] Fijalkowska D, Verbruggen S, Ndah E, Jonckheere V, Menschaert G, Van Damme P. eIF1 modulates the recognition of suboptimal translation initiation sites and steers gene expression via uORFs. Nucleic Acids Res. 2017; doi: 10.1093/nar/gkx469 28541577 10.1093/nar/gkx469 PMC5570006

[40] Cheung Y-N, Maag D, Mitchell SF, Fekete CA, Algire MA, Takacs JE, et al Dissociation of eIF1 from the 40S ribosomal subunit is a key step in start codon selection in vivo. Genes Dev. 2007;21: 1217–1230. doi: 10.1101/gad.1528307 17504939 10.1101/gad.1528307 PMC1865493

[41] Yamamoto Y, Singh CR, Marintchev A, Hall NS, Hannig EM, Wagner G, et al The eukaryotic initiation factor (eIF) 5 HEAT domain mediates multifactor assembly and scanning with distinct interfaces to eIF1, eIF2, eIF3, and eIF4G. Proc Natl Acad Sci U S A. 2005;102: 16164–9. doi: 10.1073/pnas.0507960102 16254050 10.1073/pnas.0507960102 PMC1283452

