# Supplementary material for: Translational autoregulation of BZW1 and BZW2 expression by modulating the stringency of start codon selection
Source: PLoS One. 2018 Feb 22;13(2):e0192648. doi: 10.1371/journal.pone.0192648 (PMC5823381; doi:10.1371/journal.pone.0192648)
Supplement: S1 Fig — Sequences used for generating the logograms in Fig 1. Species identifiers of the sequences used are indicated on the left. Start codons are highlighted in green. Numbers in parentheses indicate the number of sequences in each phylogenetic branch. (PDF) [file pone.0192648.s001.pdf]

S1 Fig.

BZW1 (39)

TCTTTTATGA	*Homo sapiens*
TCTTTTATGA	*Rattus norvegicus*
TCTTTTATGA	*Ovis aries*
TCTTTTATGA	*Bos taurus*
TCTTTTATGA	*Papio anubis*
TCTTTTATGA	*Sus scrofa*
TCTTTTATGA	*Ursus americanus*
TCTTTTATGA	*Mustela putorius furo*
TCTTTTATGA	*Macaca fascicularis*
TCTTTTATGA	*Oryctolagus cuniculus*
TCTTTTATGA	*Spermophilus lateralis*
TCTTTTATGA	*Ornithorhynchus anatinus*
TCTTTTATGA	*Trichosurus vulpecula*
TCTTTTATGA	*Lonchura striata domestica*
TCTTTTATGA	*Gallus gallus*
TCTTTTATGA	*Anolis carolinensis*
TTTTTTATGA	*Xenopus laevis*
TTTTTTATGA	*Xenopus (Silurana) tropicalis*
TCTTTTATGA	*Cynops pyrrhogaster*
TCTTTTATGA	*Ambystoma tigrinum tigrinum*
TCTTTTATGA	*Taeniopygia guttata*
TCTTTTATGA	*Lipochromis sp.*
TCTTTTATGA	*Monopterus albus*
TCTTTTATGA	*Poecilia reticulata*
TCTTTTATGA	*Oryzias latipes*
TCTTTTATGA	*Dicentrarchus labrax*
TCTTTTATGA	*Sparus aurata*
TCTTTTATGA	*Acipenser transmontanus*
TCTTTTATGA	*Pimephales promelas*
TCTTTTATGA	*Salmo salar*
TCTTTTATGA	*Danio rerio*
TCTTTTATGA	*Salvelinus fontinalis*
TCTTTTATGA	*Gadus morhua*
TCTTTTATGA	*Oreochromis niloticus*
TCTTTTATGA	*Gasterosteus aculeatus*
TCTTTTATGA	*Lonchura striata*
TCTTTTATGA	*Takifugu rubripes*
TCTTTTATGA	*Leucoraja erinacea*
TCTTTTATGA	*Squalus acanthias*

BZW2 (28)

AATTTTATGA	*Homo sapiens*
AATTTTATGA	*Sus scrofa*
AATTTTATGA	*Bos taurus*
AAGTTTATGA	*Squalus acanthias*
ACTTTTATGA	*Danio rerio*
AATTTTATGA	*Macaca fascicularis*
AATTTTATGA	*Canis lupus familiaris*
AATTTTATGA	*Pongo abelii*
AATTTTATGA	*Equus caballus*
AATTTTATGA	*Ovis aries*
AATTTTATGA	*Rattus norvegicus*
AATTTTATGA	*Peromyscus polionotus subgrisieus*
AATTTTATGA	*Gallus gallus*
GGTTTTATGA	*Meleagris gallopavo*
AATTTTATGA	*Anolis carolinensis*
AATTTTATGA	*Xenopus (Silurana) tropicalis*
ACTTTTATGA	*Ictalurus punctatus*
ACTTTTATGA	*Rutilus rutilus*
ACTTTTATGA	*Pimephales promelas*
ACTTTTATGA	*Oncorhynchus mykiss*
ACTTTTATGA	*Osmerus mordax*
AGTTTTATGA	*Petromyzon marinus*
AGGTTTATGA	*Eptatretus burgeri*
AATTTTATGA	*Chinchilla lanigera*
AATTTTATGA	*Abrothrix longipilis*
AATTTTATGA	*Desmodus rotundus*
AATTTTATGA	*Capra hircus*
AATTTTATGA	*Mesocricetus auratus*

Metazoa (50)

ATTTGTATGA	*Acanthoscurria gomesiana*
AACTTTATGA	*Paracentrotus lividus*
AACTTTATGA	*Strongylocentrotus purpuratus*
ATTTTTATGA	*Branchiostoma floridae*
ATTTGTATGA	*Lumbricus rubellus*
TGGTTTATGA	*Lactuca sativa*
TTGTTTATGA	*Nicotiana tabacum*
AATTTTATGA	*Plasmodium yoelii*
TATTGTATGA	*Locusta migratoria*
TATTGTATGA	*Gryllus bimaculatus*
TATTGTATGA	*Gryllus pennsylvanicus*
TTTTGTATGA	*Endeis spinosa*
TATTGTATGA	*Reticulitermes flavipes*
TTTTGTATGA	*Ixodes scapularis*
TTTTGTATGA	*Rhipicephalus appendiculatus*
TTTTGTATGA	*Rhipicephalus microplus*
GATTGTATGA	*Lysiphlebus testaceipes*
CGTTGTATGA	*Campodea fragilis*
TGTTGTATGA	*Cichorium intybus*
TATTGTATGA	*Solenopsis invicta*
TATTGTATGA	*Nasonia vitripennis*
TATTGTATGA	*Nasonia giraulti*
TATTGTATGA	*Teleopsis dalmanni*
ATTTTTATGA	*Mimulus lewisii*
TGTTGTATGA	*Acyrthosiphon pisum*
TAGTGCATGA	*Artemia franciscana*
TTTTTTATGA	*Crassostrea gigas*
TATTGTATGA	*Drosophila willistoni*
TATTGTATGA	*Drosophila ananassae*
TATTGTATGA	*Drosophila melanogaster*
TATTGTATGA	*Heliothis virescens*
TTTTTTATGA	*Capitella sp.*
TGTTGTATGA	*Myzus persicae*
TATTGTATGA	*Cochliomyia hominivorax*
TATTGTATGA	*Cochliomyia hominivorax*
TGTTGTATGA	*Daphnia_pulex*
TATTGTATGA	*Spodoptera littoralis*
TATTGTATGA	*Samia cynthia ricini*
TATTGTATGA	*Bombyx mori*
TGTTTTATGA	*Acropora palmata*
TATTGTATGA	*Spodoptera frugiperda*
TATTGTATGA	*Helicoverpa armigera*
ATTTGTATGA	*Lottia gigantea*
TTTTTTATGA	*Crassostrea virginica*
ATTTTTATGA	*Mytilus californianus*
TTTTGTATGA	*Ilyanassa obsoleta*
AGGTATATGA	*Biomphalaria glabrata*
ATTTTTATGA	*Idiosepius paradoxus*
AGGTATATGA	*Lymnaea stagnalis*
TTTTGTATGA	*Pardosa pseudoannulata*
TTTTGTATGA	*Nephila clavipes*
TGTTGTATGA	*Glycera fallax*

Plants (112)

TGGTTTATGA	*Pinus taeda*
TGGTTTATGA	*Pinus banksiana*
TGGTTTATGA	*Pinus pinaster*
TAGTTTATGA	*Cycas rumphii*
TGGTTTATGA	*Zamia vazquezii*
TGGTTTATGA	*Picea sitchensis*
TGGTTTATGA	*Picea glauca*
CAGTTTATGA	*Marchantia polymorpha*
CGTTTTATGA	*Selaginella moellendorffii*
TAGTTTATGA	*Cryptomeria japonica*
TTGTTTATGA	*Welwitschia mirabilis*
AAGTTTATGA	*Huperzia serrata*
TGGTTTATGA	*Chamaecyparis obtusa*
TGGTTTATGA	*Adiantum capillus-veneris*
TGATTTATGA	*Gametophyte rehydration*
CGGTTTATGA	*Ceratopteris richardii*
TGGTTTATGA	*Grimmia pilifera*
CAGTTTATGA	*Physcomitrella patens*
CAGTTTATGA	*Syntrichia ruralis*
TAGTTTATGA	*Ginkgo biloba*
TGGTTTATGA	*Lactuca sativa*
TGGTTTATGA	*Lactuca perennis*
TGGTTTATGA	*Lactuca virosa*
TGGTTTATGA	*Lactuca saligna*
TTGTTTATGA	*Nicotiana tabacum*
TGGTTTATGA	*Centaurea solstitialis*
TGGTTTATGA	*Centaurea maculosa*
TGGTTTATGA	*Helianthus petiolaris*
TGGTTTATGA	*Helianthus annuus*
TGGTTTATGA	*Helianthus tuberosus*
TGGTTTATGA	*Helianthus ciliaris*
TGGTTTATGA	*Helianthus argophyllus*
TGGTTTATGA	*Carthamus tinctorius*
TGGTTTATGA	*Cichorium intybus*
TGGTTTATGA	*Parthenium argentatum*
TGGTTTATGA	*Artemisia annua*
TGGTTTATGA	*Guizotia abyssinica*
TGGTTTATGA	*Barnadesia spinosa*
TTGTTTATGA	*Citrus reshni*
TTGTTTATGA	*Citrus clementina*
TTGTTTATGA	*Citrus sinensis*
TGGTTTATGA	*Populus x canadensis*
TGGTTTATGA	*Populus trichocarpa*
TGGTTTATGA	*Gossypium raimondii*
TGGTTTATGA	*Gossypium hirsutum*
TTGTTTATGA	*Ribes americanum*
TGGTTTATGA	*Vigna unguiculata*
TGGTTTATGA	*Hevea brasiliensis*
TGGTTTATGA	*Ricinus communis*
TGGTTTATGA	*Phaseolus vulgaris*
TGGTTTATGA	*Coffea canephora*
TGGTTTATGA	*Coffea arabica*
TGGTTTATGA	*Glycine max*
TGGTTTATGA	*Acacia mangium*
TGGTTTATGA	*Fagus sylvatica*
TGGTTTATGA	*Quercus robur*
TGGTTTATGA	*Quercus petraea*
TGGTTTATGA	*Castanopsis sieboldii*
TGGTTTATGA	*Jatropha curcas*
TGGTTTATGA	*Cyamopsis tetragonoloba*
TGGTTTATGA	*Eucalyptus globulus*
TGGTTTATGA	*Manihot esculenta*
TGGTTTATGA	*Carica papaya*
TGGTTTATGA	*Actinidia chinensis*
TGGTTTATGA	*Actinidia deliciosa*
TGGTTTATGA	*Glycyrrhiza uralensis*
TGGTTTATGA	*Vitis vinifera*
TGGTTTATGA	*Elaeis oleifera*
TGGTTTATGA	*Elaeis guineensis*
TGGTTTATGA	*Lotus japonicus*
TGGTTTATGA	*Arachis hypogaea*
TGGTTTATGA	*Arachis ipaensis*
TGGTTTATGA	*Ficus elastica*
TAGTTTATGA	*Lens culinaris*
TAGTTTATGA	*Lathyrus odoratus*
CGGTTTATGA	*Adonis aestivalis*
TGGTTTATGA	*Thellungiella halophila*
TGGTTTATGA	*Liriodendron tulipifera*
TGGTTTATGA	*Cucurbita maxima*
TGGTTTATGA	*Amborella trichopoda*
TGGTTTATGA	*Actaea racemosa*
TGGTTTATGA	*Musa ABB Group*
TGGTTTATGA	*Ipomoea batatas*
TGGTTTATGA	*Cucumis melo*
TGGTTTATGA	*Brassica napus*
TGGTTTATGA	*Brassica rapa*
TGGTTTATGA	*Nuphar advena*
TGGTTTATGA	*Solanum lycopersicum*
TGGTTTATGA	*Solanum melongena*
TGGTTTATGA	*Cynara cardunculus*
TGGTTTATGA	*Aristolochia fimbriata*
TGGTTTATGA	*Iris brevicaulis*
TGGTTTATGA	*Mimulus guttatus*
TGGTTTATGA	*Raphanus sativus*
TGGTTTATGA	*Raphanus raphanistrum*
TGGTTTATGA	*Theobroma cacao*
CGGTTTATGA	*Triphysaria pusilla*
TGGTTTATGA	*Capsicum annuum*
TGGTTTATGA	*Fragaria vesca*
TTGTTTATGA	*Panicum virgatum*
TTGTTTATGA	*Zea mays*
TGGTTTATGA	*Paullinia cupana*
TTGTTTATGA	*Festuca pratensis*
TTGTTTATGA	*Festuca arundinacea*
TTGTTTATGA	*Triticum aestivum*
CTGTTTATGA	*Cynodon dactylon*
TTGTTTATGA	*Brachypodium distachyon*
TTGTTTATGA	*Avena barbata*
TTGTTTATGA	*Avena sativa*
TTGTTTATGA	*Saccharum hybrid*
TGGTTTATGA	*Diospyros kaki*
TGGTTTATGA	*Arachis duranensis*

Rhodophyta (13)

TCAAAGATGG	*Grateloupia catenata*
CTCGCAATGG	*Grateloupia* livida
TCATCAATGG	Grateloupia filicina
TCAACAATGG	*Grateloupia turuturu*
GGCGTCATGG	*Dumontia simplex*
TCAGCCATGT	*Pyropia yezoensis*
GCAGCCATGT	*Pyropia haitanensis*
CGGACGATGG	*Heterosiphonia pulchra*
TCTACTATGG	*Mazzaella japonica*
CCCCCGATGG	*Gracilaria chilensis*
TTCGTCATGG	*Betaphycus philippinensis*

GCCATCATGG	*Eucheuma denticulatum*
CGCACCATGG	*Kappaphycus alvarezii*

Fungi (28)

ATATATATGT	*Spizellomyces punctatus*
TAGTTCATGA	*Phycomyces blakesleeanus*
CAGCGCATGT	*Saitoella complicate*
ATTTTAATGA	*Glomus intraradices*
CTGTGGATGT	*Heterobasidion annosum*
CAGTGCATGT	*Coprinopsis cinerea*
TAGCGGATGT	*Leucosporidium scottii*
CTGTGGATGT	*Postia placenta*
CAGTTGATGT	*Melampsora laricis-populina*
CTATACATGA	*Cunninghamella elegans*
CAGTGCATGT	*Schizophyllum commune*
TATTGTATGT	*Cryptococcus neoformans*
TTTTTAATGA	*Gigaspora margarita*
CTATACATGA	*Absidia caerulea*
ATATACATGA	*Mucor irregularis*
TATTACATGA	*Rhizopus oryzae*
CAGTTGATGT	*Cronartium ribicola*
TAGTTGATGT	*Puccinia striiformis*
TATTGTATGA	*Entomophthora muscae*
TAGTGCATGT	*Agrocybe aegerita*
CTGTGGATGT	*Leucoagaricus gongylophorus*
TAGTGGATGT	*Rigidoporus microporus*
CTGTGGATGT	*Armillaria ostoyae*
CTGTGGATGT	*Coriolopsis gallica*
CTGTGGATGT	*Sparassis latifolia*
CAGTGTATGT	*Hypsizygus marmoreus*
TGTTGCATGT	*Rhizoctonia solani*
CAGCGCATGT	*Glaciozyma antarctica*

Alveolata (5)

AATTTTATGA	*Eimeria tenella*
AATTTTATGA	*Plasmodium yoelii*
TCTTTTATGA	*Toxoplasma gondii*
TTTTTTATGA	*Cryptocaryon irritans*
TGTTTTATGA	*Symbiodinium muscatinei*

Stramenopiles (5)

TGTTGTATGA	*Sargassum vulgare*
AAGCGCATGA	*Nitzschia sp.*
GCTTGTATGA	*Chorda filum*
GCTTGTATGA	*Sargassum fusiforme*
TGGTTTATGA	*Pleurophycus gardneri*

Chlorophyta (15)

GGTTGTATGA	*Coccomyxa sp.*
CTCTGCATGA	*Prototheca wickerhamii*
TATCACATGA	*Volvox carteri*
CTTCTCATGA	*Chlamydomonas reinhardtii*
CTGTGAATGT	*Chlorella variabilis*
CTCTGCATGA	*Auxenochlorella protothecoides*
AAGCGTATGA	*Chlorella sorokiniana*
CGCTGCATGT	*Ettlia oleoabundans*
CTTTGTATGA	*Tetraselmis sp.*
CTTTGTATGA	*Tetraselmis subcordiformis*
ATTGTTATGA	*Chlamydomonas acidophila*
CCTGTTATGA	*Chlamydomonas moewusii*
TCTTGTATGA	*Botryococcus braunii*
TGTTTTATGA	*Elliptochloris marina*
GAGTTTATGA	*Caulerpa taxifolia*
