# Supplementary material for: Translational autoregulation of BZW1 and BZW2 expression by modulating the stringency of start codon selection
Source: PLoS One. 2018 Feb 22;13(2):e0192648. doi: 10.1371/journal.pone.0192648 (PMC5823381; doi:10.1371/journal.pone.0192648)
Supplement: S2 Fig — (A) The sequence of human BZW1 mRNA. (B) The sequence of human BZW2 mRNA. The main open reading frame is highlighted in yellow. The stop codon is highlighted in red. The first and second in-frame AUG codons are highlighted in light green. The out-of-frame AUG codons between the first and second in-frame AUG codons are highlighted in dark green. −3 and +4 nucleotides matching favorable initiation context are highlighted in gray, while those that are unfavorable are highlighted in magenta. (PDF) [file pone.0192648.s002.pdf]

S2 Fig.

A. *Homo sapiens BZW1*

AGGAGACACCGCCGCAGTTGCCGGTACATCGGGGATTTCTGGCTCTTTCCTCTTCGCCTTAAATTCGGGTGTCTTTT
ATGAATAATCAAAAGCAGCAAAAGCCAACGCTATCAGGCCAGCGTTTTAAAACTAGAAAAAGAGATGAAAAAGAGAG
GTTTGACCCTACTCAGTTTCAAGACTGTATTATTCAAGGCTTAACTGAAACCGGTACTGATTTGGAAGCAGTAGCTA
AGTTTCTTGATGCTTCTGGAGCAAAACTTGATTACCGTCGATATGCAGAAACACTCTTTGACATTCTGGTGGCTGGT
GGAATGCTGGCCCCAGGTGGTACACTGGCAGATGACATGATGCGTACAGATGTCTGCGTGTTTGCAGCCCAAGAAGA
TCTAGAGACCATGCAAGCATTTGCTCAGGTTTTTAACAAGTTAATCAGGCGCTACAAATACCTGGAGAAAGGTTTTG
AAGATGAAGTAAAAAAGCTGCTGCTGTTCTTGAAGGGTTTTTCAGAGTCGGAGAGGAACAAGCTAGCTATGTTGACT
GGTGTTCTTCTGGCTAATGGAACACTTAATGCATCCATTCTTAATAGCCTTTATAATGAAAATTTGGTTAAAGAAGG
AGTTTCAGCAGCTTTTGCTGTGAAGCTCTTTAAATCATGGATAAATGAAAAAGATATCAATGCAGTAGCTGCAAGTC
TTCGGAAAGTCAGCATGGATAACAGACTGATGGAACTCTTTCCTGCCAATAAGCAAAGTGTTGAACACTTCACAAAA
TATTTTACTGAGGCAGGCTTGAAAGAGCTTTCAGAATATGTTCGGAATCAGCAAACCATCGGAGCTCGTAAGGAGCT
CCAGAAAGAACTTCAAGAACAGATGTCCCGTGGTGATCCATTTAAGGATATAATTTTATATGTCAAGGAGGAGATGA
AAAAAAACAACATCCCAGAGCCAGTTGTCATCGGAATAGTCTGGTCAAGTGTAATGAGCACTGTGGAATGGAACAAA
AAAGAGGAGCTTGTAGCAGAGCAAGCCATCAAGCACTTGAAGCAATACAGCCCTCTACTTGCTGCCTTTACTACTCA
AGGTCAGTCTGAGCTGACTCTGTTACTGAAGATTCAGGAGTATTGCTATGACAACATTCATTTCATGAAAGCCTTCC
AGAAAATAGTGGTGCTTTTTTATAAAGCTGAAGTCCTGAGCGAGGAGCCCATTTTGAAGTGGTATAAAGATGCACAT
GTTGCAAAGGGGAAGAGTGTTTTCCTTGAGCAAATGAAAAAGTTTGTAGAATGGCTCAAAAATGCTGAAGAAGAATC
TGAATCTGAAGCTGAAGAAGGTGACTGAATTTTGAAACTACACCCTCAGTAAAGCAAACAGGAGTTGTAGATAAAAT
GTCATGTCTCATGTGTCCTGGTTCTTACATCTTCCTACCTCCCTGTATCAAGCATGATATAAGGGCTTTCATGGCAA
ATTTTATTTTAACTGTTTCTATGGTTGCTGGAAATGTTGGGTTTAGTTTCTAAAACCATGTTTTAAGTAGCTACAGG
AGCTATAGATTTGAATCTAATGTTGCATTAGTCTTTTCAGTTATCTTCTACCTCCTGTATTTTCTACTGTAATAATG
TAATTTAAGGCCTTCCACAATGAACAGTTCACTTTATTCCCTGGGTTTTCTATAAACAGTTTTAAGGATATGATTTG
GTTAAAAAATAATTTGTTATAAAAATTCTGTTTGCAAATTAAACTGGAAAAGTATCCAGAGTCTCAAAAGGCAATGA
TTTGTGAGATAATATGGCATGCCCGGAGCCCTGCTCATCAATGAAAAACCCATATGTAATAATCGAATTCATTTAAC
ATGAATCTTGAGTACGTGGACCATTGCTTGCATGTTAACTTTTTGTTTTGTTTTGTTTTGTTTTGTTTTGCATTTTT
AACTCCAGATATCCTAAAGCTCAATTGTTTGGTCTCTGGTTTTCATCCTTAGAGAAGCCATGGAGAACAGACTTGAA
AAGTTTAGGAAATCATAATGTGGCAGAGGTGGTGGGAAGAAGAAAGTTGAGCTTTTTCCCCTTGAGAAACTTCTGCA
TTTAGTTTCTATCTTTCCAGGCAAAACAAATGGGTATTCTTTTCATACAACCATTTTCAAATGAACCTTAGAAAAGT
CTTAACATTTAAGGTATTTTATGCACAGAATACACTTAGATTGATAGGAAAGAACTCGTAATGGAGTTTGAGTAAAG
AAAATGACTGATGTACTAAACCCAGTAAAAATTGTTGAAAATGTTAAAGGTCAGCATGTTCTAATTGGGAATCTAGA
TATAGCTTAGATTTCCTATTGGCTTAGAGTATTTGCTATAACAAATGAAGTGCAATGACAATTATATATTCCTACTC
GGTCATACTGGACTGGCTTCGTTCTCTTAATATACTCAGTAATGACTCAAGCCTCTGGCTATTAACATACCCTAGTT
GCCGTTTTTTAATTGCCATGAGCCAAATACTTCTTGGTATACAATTGATCCATTTATTTTAATGGCTGCCTTTTCAT
TTTCATCTTTTCTTGCTGCTACCCATCTATGTATGTAGTCATTGGGGGGAAAATGTAGCCACATTTTTTATGGGAAG
ACTTTGTGTTAAAAGTGAACATTTTGAAGGTTTTTAACTGGTGAAACTAGCCTGGAATAATGCCACCAGAGACTGAG
TGGAAATCGCCCCTTTTGAAGGTGCCATTCTTATGAGCCAAAAGTTTGTCATTTAAAAGTTCATTTTGAGGGAATAA
CATGTAATATAATTTGAAATAAAGGTATAGTAACCTTAAAAAGAACATTATAACTGATTGTTGTGAATGGGGTGAAT
TTGTTAAAATGAGTAACTTTGATAAAGTTTTTCATGCACAGGCAAAATGTATTCACTAGATTTCTACGTAGTGATCT
GCTTTTACTTTGTAATTTGTAGTTCTCAAAAGACTTTTTTTTAAAAAAATAAAGTCCATACTTACACTTAAAAAAAA
AAAAAAAAAAA

B. *Homo sapiens BZW2*

CTTCACTCCTCCATTGTCTGCCGCCACTGCTGCTGCTGCTGCTGCTGCCGCTGCTGCTGCACGAATCGCCGCAGCCC
CCAGCCTTGCGCGTCGTCGCTACCTCCTCGGACAGAAATTTTATGAATAAGCATCAGAAGCCAGTGCTAACAGGCCA
GCGGTTCAAAACTCGGAAAAGGGATGAAAAAGAGAAATTCGAACCCACAGTCTTCAGGGATACACTTGTCCAGGGGC

TTAATGAGGCTGGTGATGACCTTGAAGCTGTAGCCAAATTTCTGGACTCTACAGGCTCAAGATTAGATTATCGTCGC
TATGCAGACACACTCTTCGATATCCTGGTGGCTGGCAGTATGCTTGCCCCTGGAGGAACGCGCATAGATGATGGTGA
CAAGACCAAGATGACCAACCACTGTGTGTTTTCAGCAAATGAAGATCATGAAACCATCCGAAACTATGCTCAGGTCT
TCAATAAACTCATCAGGAGATATAAGTATTTGGAGAAGGCATTTGAAGATGAAATGAAAAAGCTTCTCCTCTTCCTT
AAAGCCTTTTCCGAAACAGAGCAGACAAAGTTGGCGATGCTGTCGGGGATTCTGCTGGGCAATGGCACCCTGCCCGC
CACCATCCTCACCAGTCTCTTCACCGACAGCTTAGTCAAAGAAGGCATTGCGGCCTCATTTGCTGTCAAGCTTTTCA
AAGCATGGATGGCAGAAAAAGATGCCAACTCTGTTACCTCGTCTTTGAGAAAAGCCAACTTAGACAAGAGGCTGCTT
GAACTCTTTCCAGTTAACAGACAGAGTGTGGATCATTTTGCTAAATACTTCACTGACGCAGGTCTTAAGGAGCTTTC
CGACTTCCTCCGAGTCCAGCAGTCCCTGGGCACCAGGAAGGAACTGCAGAAGGAGCTCCAGGAGCGTCTTTCTCAGG
AATGCCCGATCAAGGAGGTGGTGCTTTATGTCAAAGAAGAAATGAAGAGGAATGATCTTCCAGAAACAGCAGTGATT
GGTCTTCTGTGGACATGTATAATGAACGCTGTTGAGTGGAACAAGAAGGAAGAACTTGTTGCAGAGCAGGCTCTGAA
GCACCTGAAGCAATATGCTCCCCTGCTGGCCGTGTTCAGCTCCCAAGGCCAGTCAGAGCTGATCCTCCTCCAGAAGG
TTCAGGAATACTGCTACGACAACATCCATTTCATGAAAGCCTTTCAGAAGATTGTGGTTCTCTTTTATAAAGCTGAT
GTTCTGAGCGAAGAAGCAATACTGAAATGGTATAAGGAAGCACATGTTGCTAAAGGCAAAAGTGTTTTTCTTGACCA
GATGAAGAAATTTGTTGAGTGGTTACAAAATGCAGAAGAAGAATCCGAATCGGAAGGTGAGGAAAATTAAATGGCTC
AACAAGCACAATACCTAGGTTACCACACACCACTTTTTGATTGGGAATGCTGAACCATTTGAGAAGAGAAACTTGGC
TTCTGTTTTCGCAAAGGAAAAAAAAAAATAGGATAGGCTTCCCTTGTGCAGAGGGAGAAATGGTTTTGTTTTTGTTTT
GTTTTTAAATGGAGCCCTGAGGCATCAGCTATTATACTTGGGACTCTACCTCTCACTCACTATATGCTAACTTAAAG
CCATTCAACAAGGAGTCAAGTAGATCTGAAATTAAATACTCAACAGACTCCTCCTTTTTTAGCTGTATTTTTCAGGT
ACTGTGTGGTGACCGCCCCACTGGTGTCTATTACAGGCCACTTTGGTAGTTGTGTATCTGCTCATGTATGTGATTTG
ACAAACCAGTTTTTTAAAATAAATGGCTTTTTAAAAATCTGGAAAAAAAAAAA
