# Supplementary material for: Translational autoregulation of BZW1 and BZW2 expression by modulating the stringency of start codon selection
Source: PLoS One. 2018 Feb 22;13(2):e0192648. doi: 10.1371/journal.pone.0192648 (PMC5823381; doi:10.1371/journal.pone.0192648)
Supplement: S1 Table — (PDF) [file pone.0192648.s003.pdf]

S1 Table

Oligo #	Oligo name	Sequence (5' – 3')
1	BZW1FL/S1	GTAGTCTTTTATGACCG
2	BZW1FL/A1	GATCCGGTCATAAAAGACTACTGCA
3	BZW2FL/S1	GTAGAATTTTATGACCG
4	BZW2FL/A1	GATCCGGTCATAAAATTCTACTGCA
5	BZW1/S1	CGCGCTAGCTAGTCTTTTATGAATAATCAAAAGCAGCAAAAGC
6	BZW1AUGB/S2	CGCGCTAGCTAGGCCACCATGAATAATCAAAAGCAGCAAAAGCCAACGCT
7	BZW1/A1	CGCTCTAGACACTCAGTCACCTTCTTCAGCTTCAGATTC
8	BZW2/S1	CGCGCTAGCTGAAATTTTATGAATAAGCATCAGAAGCCAGTG
9	BZW2AUGB/S2	CGCGCTAGCTGAGCCACCATGAATAAGCATCAGAAGCCAGTGCTAAC
10	BZW2/A1	CGCTCTAGACACTTAATTTTCCTCACCTTCCGATTCGGATTC
